# What is the level of nutrition literacy of Saudi adolescents? A national wide exploratory cross-sectional study

**DOI:** 10.3389/fnut.2022.1113910

**Published:** 2023-01-12

**Authors:** Khlood Bookari

**Affiliations:** ^1^Department of Clinical Nutrition, Faculty of Applied Medical Sciences, Taibah University, Medina, Saudi Arabia; ^2^National Nutrition Committee (NNC), Saudi Food and Drug Authority (Saudi FDA), Riyadh, Saudi Arabia

**Keywords:** nutrition literacy, Arab world, kingdom of Saudi Arabia, Saudi, adolescents

## Abstract

**Background:**

Despite being a prolific research topic, studies evaluating nutrition literacy in the Arab world are scant and still nonexistent in the Kingdom of Saudi Arabia. Therefore, a national study was launched with the aim to (1) assess nutrition literacy among Saudi adolescents aged 10–19 years old and (2) determine its correlates.

**Methods:**

A cross-sectional study was launched across all Saudi provinces between 29 April and 6 June 2022, enrolling a convenience sample of 2,115 adolescents (mean age = 16.8 ± 2.5; girls: 48.7%). An online self-administered questionnaire was disseminated to eligible participants to collect relevant data.

**Results:**

Study findings showed that nearly half of the adolescents (44.6%) had poor nutrition literacy. The male gender doubled the risk of adolescents having poor nutrition literacy (OR = 2.0, CI = 1.5-2.5, *p* < 0.001). Northern border residents were 14 times more likely to be nutritionally illiterate, in contrast to adolescents residing in Riyadh (OR = 14.0, CI = 7.3–28.0, *p* < 0.001). Adolescents were more likely to be nutritionally illiterate if they were underweight or overweight (OR = 2.7, CI = 1.6–4.7, *p* < 0.001; OR = 2.2, CI = 1.7–2.9, *p* < 0.001). School students had 2 times more risk of having poor nutrition literacy, in contrast to those who were enrolled in universities (OR = 1.8, CI = 1.4–2.4, *p* < 0.001). Nutrition illiteracy was 4 times higher among adolescents having caregivers else than their parents (OR = 3.9, CI = 2.2–6.9, *p* < 0.001). Parental education level also contributed to determining adolescents' nutrition literacy status.

**Conclusions:**

It has become essential to courage the development of supportive school environments in Saudi Arabia to promote nutrition education and improve adolescents' nutrition literacy. Without a doubt, this calls for taking a holistic approach on the part of education and health ministries, nutrition professionals, educators, parents, and, most importantly, the teenagers themselves, who must have the desire and motivation to learn.

## 1. Introduction

Research debates on nutrition literacy topics have been receiving prominence over the years. Nutrition literacy extends beyond just people reading about nutrition; it also includes numeracy and cognitive abilities that shape healthy food selections and eating behaviors (1). Nutrition literacy is “the degree to which people have the ability to obtain, process and understand basic diet information, as well as the tools needed to make appropriate nutrition decisions” (2). Adequate nutrition literacy requires an individual not only to read well but also to understand health and nutrition concepts and to have basic quantitative skills (defined as numeracy: “the ability to use and understand numbers in daily life, including the ability to read and interpret nutrition information” (3). People without these skills may have difficulty understanding concepts of healthful diets, reading nutrition information, and measuring portion size (3). Functional nutrition literacy (FNL), interactive nutrition literacy (INL), and critical nutrition literacy (CNL) are the three nutrition literacy levels that have been identified over time. FNL stands for functional numeracy literacy, or the ability to apply basic literacy abilities, such as reading and comprehending food labels and understanding the fundamentals of dietary guidelines. INL includes more sophisticated literacy abilities, such as the capacity to communicate effectively with nutrition counselors on a cognitive and interpersonal level, as well as a desire to find and use accurate nutrition information to change one's eating habits and behavior. CNL refers to the ability to analyze nutrition information and recommendations critically as well as the willingness to take part in initiatives to eliminate nutritional barriers from a personal, social, and global perspective (4). It has been noticed that people and communities with higher nutrition literacy are able to comprehend nutrition information and, as a result, adhere to a healthy diet. Since they are more knowledgeable about the relationship between a poor diet and disease, nutrition literacy can help lower the disease burden and lessen economic and health disparities in underprivileged areas (5). Improper eating habits are one of the reasons that worldwide public wellness is approaching a tipping point. One attempt is made to improve a poor diet through nutrition literacy programs (6). Nutrition literacy is growing in importance in the management, treatment, and prevention of non-communicable diseases (NCDs) (7). This is particularly important given that every year, NCDs cause the deaths of 41 million people worldwide (8). As food preferences and eating habits are founded early in life, children and adolescents must equip adequate nutrition literacy levels to guide appropriate eating behaviors (9). The initiative to promote nutritional literacy is an active endeavor to educate the general population, and adolescents in particular, on the value of health. Nutrition literacy programs are focused on the effort to learn more about healthy lifestyle, understand the motivations for choosing to live a healthy lifestyle, take an active role in helping others, and critique the sources of food consumed (10).

Adolescence, according to the World Health Organization (WHO), is the period between the ages of 10 and 19. Adolescents are a transitional population, sharing some nutrition-related issues with children and some with adults (11). Adolescence is the stage of life during which, after infancy, growth occurs at the fastest rate, being oversensitive to nutrition deficiencies (12). Adolescent nutrition is critical for compensating for childhood deficiencies and should include nutrients required to meet the demands of physical and cognitive growth and development, provide adequate stores of energy for illnesses and pregnancy, and prevent adults onset of nutrition-related diseases (11). Additionally, adolescents desire to grow self-reliant and forge their identity in all aspects, including their food choices and eating habits. And hence, nutrition illiteracy might cause adolescents to shoulder poor nutrition outcomes and health vulnerabilities when undesirable choices occur (13). Nutrition literacy had been frequently linked to eating behaviors, dietary diversity, nutrient adequacy, food label use, and school performance among multiple demographics, particularly adolescents (14–18).

There are 1.3 billion adolescents worldwide as of today, making up 16% of the world's population (19). Saudi Arabia, like other nations, is witnessing a youth revival, with 20% of its population being adolescents between the ages of 10 and 19 (20). Furthermore, it was estimated that, by 2030, Saudi Arabia will have a 12% increase in the school-age population (21). Effective public health initiatives like children's immunizations and healthcare providers have generally been successful in Saudi Arabia in reducing the burden of communicable diseases and infant mortality, respectively. Non-communicable diseases (NCDs) largely account for the current disease burden; hence adult-specific NCDs have taken center stage on the healthcare and health research agendas. The key modifiable risk factors, such as tobacco use, excessive alcohol use, unhealthy diets, and insufficient physical activity, start much earlier in life during adolescence, so the emphasis should be on the adolescent period and prevention of these risk factors as well as promotion of protective factors (11). Late adolescents and youth are preparing to or have recently experienced independent living as they go from adolescence to adulthood. As a result, adolescents are often given additional responsibilities for organizing, choosing, and cooking meals than younger generation. Because of this, the level of knowledge and skills that this age group has regarding food and nutrition may be able to assist them in navigating the complex and multifaceted factors influencing their dietary practices, which may have a significant impact on their eating patterns and health in the future (22).

Adolescents' poor eating habits in Saudi Arabia are a major public health concern, and during the COVID-19 restriction period, these unhealthy habits were made worse (11). In 2021, nearly 24 and 41% of Saudi male and female adolescents were obese, respectively (23). Rapid urbanization and lifestyle changes brought about by Saudi Arabia's flourishing economy have contributed substantially to sedentary behavior, western patterns of diets, and the country's rising rates of overweight and obesity (24). Obesity among Saudi adolescents was also observed to be linked to unfavorable eating habits, including daily consumption of soft drinks (25). Along these, unhealthy dietary patterns were reported among Saudi adolescents; manifested by fast food consumption, skipping breakfast, and low fruit and vegetable consumption (26). Adolescents are thought to be the predominant consumers of fast food. The extensive availability and ubiquity of fast-food restaurants, the taste preferences, the comparatively low cost of large serving sizes, and convenience; all contribute to this. A previous study in Saudi Arabia showed that 85% of adolescent participants preferred fast food to home cooking (27). Despite these facts, researchers and decision-makers in the discipline of nutrition typically concentrate on children, women, and the elderly, overlooking the “neglected” adolescents (28).

The majority of the interventions, so far, have either been directed toward pregnant women or, to a minor extent, lactating women or children aged 0 to 5 years. Adolescents haven't yet, however, received much attention from nutrition-related programs. Adolescent-specific programs are severely lacking in the Arab region. Lack of age and sex-disaggregated data on the health and nutritional status of adolescents at the national level is one of the main underlying causes of the absence of policies and programs for improving their health and nutritional status. Adolescents are often neglected in public health initiatives due to a lack of qualified healthcare professionals and facilities that can cater to their unique demands (11). According to the available evidence, no studies have so far been done to assess nutrition literacy among Saudi adolescents. This, coupled with the most recent review's warning on the need to address the nutrition literacy topic in the Arab region (28), motivates us to conduct the current study. Therefore, the author launched this national study to be the first of the region's kind to (1) assess nutrition literacy among Saudi adolescents aged 10–19 years old and (2) determine its correlates.

## 2. Methods and materials

### 2.1. Study design and participants' recruitment

This study followed a cross-sectional design and the convenience snowballing method. It was launched between 29 April and 6 June 2022. An online-based self-administered questionnaire consisting of multiple parts was disseminated to eligible participants to meet the study aims. To increase the sample representation and generalizability of the results, adolescents were recruited from almost all of Saudi Arabia's provinces: Riyadh, Makkah, Madinah, Eastern province, Northern borders, Jawf, Tabuk, Ha'il, Qasim, Bahah, Asir, and Najran. The research team attempted to advertise the study on different social media platforms with the aid of the author's academic networks and scholarly connections. To encourage participation, participants and their guardians were informed that this study is intended to be the first stepping stone forward in actively advocating for nutrition education in Saudi Arabian schools. Overall, in this study, the author enrolled a total convenient sample of 2,115 adolescents aged 10–19 years old.

### 2.2. Adolescents' eligibility

The adolescent should be 10–19 years old and have Saudi nationality to be regarded for inclusion. This age range was determined based on the WHO's claim that the adolescence stage is a life period ranging from 10–19 years old (29). Additionally, to ensure a more diverse sample for this study, one adolescent child per household was enrolled.

### 2.3. Covariates

#### 2.3.1. Demographic information

As designed by nutrition experts, the questionnaire contains the following inquiries to cover the adolescents' demographic characteristics in relevance to the study aims: age, gender, current residency, education level, maternal education level, paternal education level, adolescents' working status, and the primary caregiver(s) (mother, father, other caregivers, and living alone). Adolescents were asked to self-report their body weight and height; consequently, adolescents were classified as being underweight, normal-weight, overweight, or obese per the WHO criteria on body weight classification (30).

#### 2.3.2. Nutrition education in school settings

In line with the study objectives, adolescents were asked if they typically receive nutrition education in their schools' curriculum, with the type of school they were enrolled in (public school, private school). The adolescents had the option of a binary response (Yes, I receive nutrition education in my school; No, I don't). This could give insights into the urge of starting intervening in adolescents' nutrition knowledge and skills in school settings.

### 2.4. Outcome variable: Nutrition literacy

To assess adolescents' nutrition literacy (total nutrition literacy: TNL), the author used the valid 22-items Adolescent Nutrition Literacy Scale (ANLS) (31) (Refer to Table 1). ANLS evaluated three components of TNL: Functional NL (FNL) (7 questions), Interactive NL (INL) (6 questions), and Critical NL (CNL) (9 questions). FNL reflects the capacity to understand nutrition information. INL refers to the communication and interactive skills required to attain nutrition information. CNL reflects the ability to critically evaluate nutrition information (4). Each question in the ANLS has a score ranging from 1 to 5 (5-points Likert scale: strongly disagree to strongly agree). A higher score indicates better NL. The questionnaire has an overall score range from 22 to 110. The median scores, or second quartiles, of the study participants were used to determine the cutoffs. The adolescents were classified as having adequate or poor nutrition literacy based on the observed median scores, which were 67.0, 24.0, 17.0, and 28.0, respectively, for the TNL, FNL, INL, and CNL. Thus, an overall score of less than 67.0 indicated poor nutrition literacy, and a score of 67.0 and above (over 110) was an indication of adequate nutrition literacy among adolescents in this study (31).

**Table 1:** The adolescent nutrition literacy scale (ANLS).

**Question**	**Dimension**	**Min-Max score**
1. I find that the language used by nutrition, health and food experts is difficult to be understood.	Functional Nutrition Literacy (FNL)	Strongly disagree = 5 (max score) to Strongly agree = 1 (min score) (reverse-coded question)
2. I find it difficult to understand the jargon (technical words) used by nutrition, health and food experts	FNL	Strongly disagree = 5 (max score) to Strongly agree = 1 (min score) (reverse-coded question)
3. When I read information about nutrition, food, or diet, I find it difficult to understand	FNL	Strongly disagree = 5 (max score) to Strongly agree = 1 (min score) (reverse-coded question)
4. I find it difficult to know how I should change my diet when I get dietary advice from the doctor, nurse or the like	FNL	Strongly disagree = 5 (max score) to Strongly agree = 1 (min score) (reverse-coded question)
5. When I read information about nutrition food or diet, I need someone to help me understand it.	FNL	Strongly disagree = 5 (max score) to Strongly agree = 1 (min score) (reverse-coded question)
6. I am not familiar with World Health Organization (WHO) recommendations for daily intake of fruits and vegetables.	FNL	Strongly disagree = 5 (max score) to Strongly agree = 1 (min score) (reverse-coded question)
7. When I read an article about nutrition, food, or diet, I find words that I don't know	FNL	Strongly disagree = 5 (max score) to Strongly agree = 1 (min score) (reverse-coded question)
8. I have gathered information about diet from various sources that I think is relevant for me.	Interactive Nutrition Literacy (INL)	Strongly agree = 5 (max score) to strongly disagree = 1 (min score)
9. I use the internet when I am looking for information about nutrition such as diet.	INL	Strongly agree = 5 (max score) to strongly disagree = 1 (min score)
10. I discuss about diet with my friends, family and relatives	INL	Strongly agree = 5 (max score) to strongly disagree = 1 (min score)
11. I have changed my eating habits based on the information about diet that I have gathered.	INL	Strongly agree = 5 (max score) to strongly disagree = 1 (min score)
12. I often read material about what constitutes a balanced diet	INL	Strongly agree = 5 (max score) to strongly disagree = 1 (min score)
13. I readily take the initiative to discuss with dietary experts (for example a doctor, nurse or the like) about healthy eating	INL	Strongly agree = 5 (max score) to strongly disagree = 1 (min score)
14. I would readily get involved in political issues targeted at improving people's diet.	Critical Nutrition Literacy (CNL)	Strongly agree = 5 (max score) to strongly disagree = 1 (min score)
15. I am willing to take an active role in measures aimed at promoting a healthier diet at my school.	CNL	Strongly agree = 5 (max score) to strongly disagree = 1 (min score)
16. I expect my school to serve healthy food.	CNL	Strongly agree = 5 (max score) to strongly disagree = 1 (min score)
17. I try to influence others (for example my family and friends) to eat healthy food.	CNL	Strongly agree = 5 (max score) to strongly disagree = 1 (min score)
18. It is important for me that the school canteens have a good selection of healthy food.	CNL	Strongly agree = 5 (max score) to strongly disagree = 1 (min score)
19. I tend to be influenced by the dietary advice I read in newspapers, magazines or elsewhere	CNL	Strongly disagree = 5 (max score) to Strongly agree = 1 (min score) (reverse-coded question)
20. I tend to be influenced by the dietary advice I get from my family, friends.	CNL	Strongly disagree = 5 (max score) to Strongly agree = 1 (min score) (reverse-coded question)
21. I believe that the media's presentation of scientific findings about nutrition, diet, food is correct.	CNL	Strongly disagree = 5 (max score) to Strongly agree = 1 (min score) (reverse-coded question)
22. When I read information about nutrition, diet or food it is important to me that it is based on scientific evidence.	CNL	Strongly agree = 5 (max score) to strongly disagree = 1 (min score)

The used questionnaire underwent a face validity (or “logic validity”) review to confirm its accuracy. Each survey question was evaluated independently by an expert panel constituted of a public health nutritionist and four registered dietitians. To assure that it included crucial elements of nutrition literacy, such as the capacity to perform fundamental tasks, interact with others, and form sound judgments, the questionnaire was double-checked. A statistician (an APD and an authority in question design) reviewed the survey for common errors (e.g., leading, confusing, or double-barreled questions). First, the study's pilot respondents were two researchers (Nutritionists) from Taibah University's Department of Applied Medical Science (TU). Then ten adolescents between the ages of 10 and 19.

### 2.5. Ethical approval of the study protocol

The ethical committee at Taibah University, Saudi Arabia, approved the current study protocol while adhering to the guidelines laid down in the Helsinki Declaration at all study stages. The study participants provided their informed consent electronically before filling out the online survey. For adolescent participants, parents or official caregivers need to give consent for the adolescent to participate in the study. To start participating in filling out the questionnaire, consent is required from both parents/guardians and the adolescent. If any of the parties, whether parents/guardians or adolescents themselves, do not agree, the questionnaire will be terminated immediately. The participation was voluntary with no penalty for refusal or withdrawal.

### 2.6. Statistical analysis

The author performed the statistical analysis using the Statistical Package of Social Sciences Software (SPSS) (Version 25.0. IBM Corp: Armonk, NY, USA). A “weighting” variable was created to enhance the representation of the study sample according to adolescents' gender and residency. Respondents' characteristics were presented as frequencies (percentages) for categorical variables, while means ± standard deviation (SD) for continuous variables. The adolescence stages were classified per the WHO criteria (24): early adolescence (10–13 years old), middle adolescence (14–16 years old), and late adolescence (17–19 years old). Continuous variables' normal distribution was checked using the Shapiro-Wilk test. The Mann-Whitney *U* test was used to detect mean differences between study variables composed of two groups (such as gender). Chi-squared test (χ2) was used to determine associations between study variables (the covariates, and the outcome variable: nutrition literacy). In addition, the backward stepwise method of binary logistic regression was used to identify the determinants of adolescents' nutrition literacy. A *p*-value of 0.05 and below was considered significant for all analytical tests.

## 3. Results

### 3.1. Demographic characteristics of adolescents

A total of 2,115 Saudi adolescents were included in this study and their data were considered for analysis. Among all adolescents, 48.7% were girls. The mean age ± SD of the overall sample was 16.8 ± 2.5, in which male adolescents were younger (16.0 ± 3.0) than their female counterparts (17.0 ± 2.0), *p* < 0.001. When stratified per their age, the majority of the adolescents (70.3%) were in their late adolescence stage (17–19 years old), whereas the remaining (29.7%) were young (10–13 years old) or middle-aged (14–16 years old) adolescents. Adolescents were recruited from almost all Saudi provinces per the following distribution: Riyadh (8.1%), Makkah (8.6%), Madinah (7.8%), Eastern province (10.7%), Northern borders (14.6%), Jawf (5.1%), Tabuk (6.7%), Ha'il (6.3%), Qasim (6.0%), Bahah (8.2%), Asir (9.0%), and Najran (8.9%). Furthermore, 38.6% of the adolescents were overweight or obese. Regarding their education level, just 0.5% of the adolescents had no formal education, while the remaining were either attending schools (59.7%) or universities (39.7%). As for their parents' highest education level, 85% of the mothers had a school or university education level, compared to 92.0% of the fathers. Most adolescents (68.8%) reported having both parents as primary caregivers. Furthermore, around one-quarter (23.0%) of the adolescents were working (Table 2).

**Table 2:** Demographic, general characteristics and the BMI of adolescents.

**Variables**	**Overall** **(*N* = 2,115)**		**Girls** **(*n =* 1,030)**		**Boys** **(*n =* 1,085)**		***p*-value**

	***N***	**%**	***n***	**%**	***n***	**%**	
**Adolescence stage**							<0.001^*^
Early adolescence (10–13 years old)	320	15.1	52	5.0	268	24.7	
Middle adolescence (14–16 years old)	308	14.6	230	22.3	79	7.3	
Late adolescence (17–19 years old)	1487	70.3	748	72.7	738	68.0	
**Current residency**							<0.001^*^
Riyadh	171	8.1	83	8.0	88	8.1	
Makkah	182	8.6	78	7.6	103	9.5	
Madinah	165	7.8	85	8.3	79	7.3	
Eastern Province	226	10.7	62	6.0	164	15.1	
Northern borders	309	14.6	27	2.6	283	26.0	
Jawf	107	5.1	107	10.4	0	0.0	
Tabuk	142	6.7	97	9.5	44	4.1	
Ha'il	134	6.3	134	13.0	0	0.0	
Qasim	126	6.0	99	9.6	27	2.5	
Bahah	174	8.2	80	7.8	94	8.7	
Asir	191	9.0	84	8.1	108	9.9	
Najran	188	8.9	94	9.1	94	8.7	
**Weight status**							<0.001^*^
Underweight	69	3.3	27	2.6	42	3.9	
Normal-weight	1,229	58.1	801	77.8	429	39.5	
Overweight	810	38.3	196	19.0	614	56.5	
Obese	7	0.3	5	0.5	2	0.1	
**Adolescent's education level**							<0.001^*^
Not attending school	11	0.5	10	0.9	1	0.2	
Elementary school level	275	13.0	33	3.2	243	22.4	
Intermediate school level	411	19.4	155	15.1	256	23.6	
Secondary school level	578	27.3	350	34.0	228	21.0	
University level	840	39.7	482	46.8	357	32.9	
**Maternal education level**							<0.001^*^
Illiterate	318	15.0	172	16.7	146	13.4	
Elementary school level	192	9.1	84	8.2	108	10.0	
Intermediate school level	546	25.8	224	21.8	322	29.6	
Secondary school level	276	13.1	153	14.8	123	11.3	
University level	783	37.0	397	38.5	386	35.6	
**Paternal education level**							<0.001^*^
Illiterate	168	8.0	54	5.3	114	10.5	
Elementary school level	187	8.9	65	6.3	122	11.3	
Intermediate school level	305	14.4	63	6.1	242	22.3	
Secondary school level	507	24.0	334	32.4	173	15.9	
University level	948	44.8	514	49.9	434	40.0	
**Primary caregiver**							<0.001^*^
Both parents	1456	68.8	786	76.3	670	61.7	
The father	59	2.8	18	1.8	41	3.8	
The mother	380	18.0	138	13.4	242	22.3	
Others	160	7.6	29	2.8	131	12.1	
None (living alone)	60	2.8	59	5.7	1	0.1	
**Currently working**							<0.001^*^
No	1,630	77.1	936	90.9	694	64.0	
Yes	485	22.9	94	9.1	391	36.0	

^*^Significant at *p*-value <0.001 for χ2 test.

### 3.2. Nutrition education in Saudi school settings

**Figure 1:**
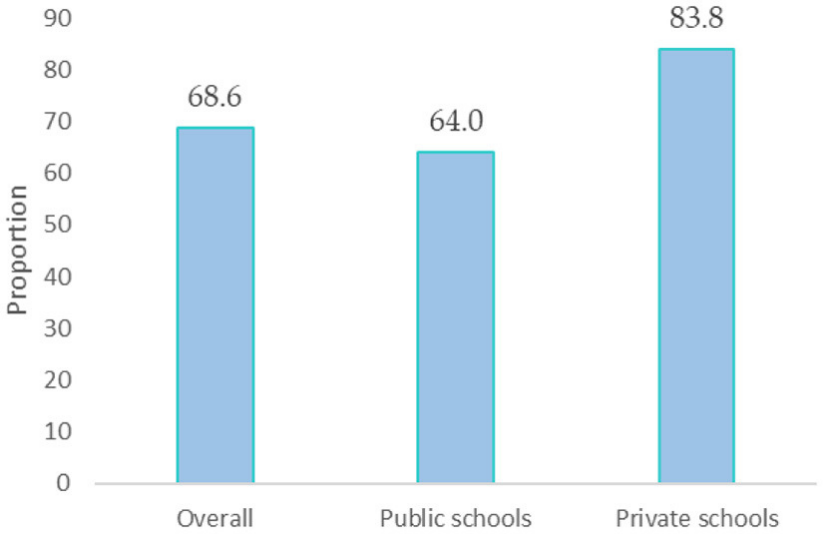
The proportion of adolescents not receiving nutrition education in school's curriculum.

The author asked school students (*n* = 1,264) to report the school type they were attending and whether they receive nutrition education in their school's curriculum. Overall, 68.6% of the students were not receiving nutrition classes in their schools, particularly those studying at private schools (83.8%), in contrast to their counterparts enrolled in public schools (64.0%), *p* < 0.001 (Figure 1).

### 3.3. Adolescents' nutrition literacy status

Overall, around 45.0% of adolescents were observed to have inadequate nutrition literacy levels (TNL). As per its components, 44.2% had functional nutrition illiteracy, 45.3% had inadequate INL, and 41.8% had poor scores in CNL. More boys than girls had poor TNL (60.6 vs. 27.8%), INL (56.3 vs. 33.8%), and CNL (49.0 vs. 34.2%), all *p-*values < 0.001 (Table 3).

**Table 3:** The status of adolescents' nutrition literacy, overall and by adolescents' gender.

	**Overall** **(*N* = 2,115)**		**Girls** **(*n =* 1,030)**		**Boys** **(*n =* 1,085)**		***p*-value**

	***N***	**%**	***N***	**%**	***N***	**%**	
**TNL** ^a^							<0.001^*^
Poor	944	44.6	286	27.8	658	60.6	
Adequate	1,171	55.4	744	72.2	427	39.4	
**FNL** ^b^							0.07
Poor	934	44.2	434	42.2	500	46.1	
Adequate	1,181	55.8	596	57.8	585	53.9	
**INL** ^c^							<0.001^*^
Poor	959	45.3	348	33.8	611	56.3	
Adequate	1,156	54.7	682	66.2	474	43.7	
**CNL** ^d^							<0.001^*^
Poor	884	41.8	352	34.2	531	49.0	
Adequate	1,231	58.2	678	65.8	554		

^a^Total Nutrition Literacy; ^b^Functional Nutrition Literacy; ^c^Interactive Nutrition Literacy; ^d^Critical Nutrition Literacy; ^*^significant at *p*-value <0.001 for χ2 test.

### 3.4. The correlations associated with the adolescents' nutrition literacy status

Table 4 displays the relationships between study variables and adolescents' nutrition literacy. Considering adolescents' age, young adolescents had mostly poor nutrition literacy (53.9%), which exceeds the prevalence reported among middle-aged (48.2%) and older adolescents (41.9%), *p* < 0.001. Nutrition illiteracy was observed among 6 out of 10 boys, in contrast to 27.8% of girls, *p* < 0.001. Accounting for their residence, poor nutrition literacy scores were prevalent the most among the Northern province residents (91.4%), exceeding that reported among all other provinces, *p* < 0.001. Of relevance, more than half (60.1%) of overweight and underweight adolescents were nutritionally illiterate, *p* < 0.001 (Table 4).

**Table 4:** The correlations associated with the adolescents' nutrition literacy status.

**Correlates**	**Nutrition literacy**		***p*-value**
	**Poor** *N* = **944**	**Adequate** *N* = **1,171**	
	***N*** **(%)**	***N*** **(%)**	
**Adolescence stage**			<0.001 ^*^
Early adolescence (10–13 years old)	172 (53.9)	147 (46.1)	
Middle adolescence (14–16 years old)	149 (48.2)	160 (51.8)	
Late adolescence (17–19 years old)	623 (41.9)	864 (58.1)	
**Gender**			<0.001^*^
Female	286 (27.8)	744(72.2)	
Male	658 (60.6)	428 (39.4)	
**Residency**			<0.001^*^
Riyadh	77 (45.3)	93 (54.7)	
Makkah	70 (38.8)	111 (61.2)	
Madinah	72 (43.6)	93 (56.4)	
Eastern Province	120 (52.5)	106 (47.5)	
Northern Province	283 (91.4)	27 (8.6)	
Jawf	53 (50.0)	53 (50.0)	
Tabuk	44 (31.2)	98 (68.8)	
Ha'il	0 (0.0)	134 (100.0)	
Qasim	48 (38.0)	78 (62.0)	
Bahah	45 (25.5)	130 (74.5)	
Asir	38 (19.9)	154 (80.1)	
Najran	94 (50.2)	94 (49.8)	
**Weight status**			<0.001^*^
Underweight	41 (60.1)	27 (39.9)	
Normal weight	414 (33.7)	816 (66.3)	
Overweight	487 (60.1)	323 (39.9)	
Obese	2 (27.6)	5 (72.4)	
**Adolescents'**			<0.001^*^
**education level**			
Not attending school	2 (19.4)	9 (80.6)	
School level	664 (52.5)	601 (47.5)	
University level	278 (33.1)	562 (66.9)	
**Maternal**			<0.001^*^
**education level**			
Never attend school	41 (13.1)	276 (86.9)	
School level	462 (48.5)	523 (51.5)	
University level	411 (52.4)	372 (47.6)	
**Paternal**			<0.001^*^
**education level**			
Never attend school	32 (18.8)	137 (81.2)	
School level	460 (46.1)	538 (53.9)	
University level	452 (47.7)	496 (52.3)	
**Primary caregiver**			<0.001^*^
Both parents	561 (38.5)	896 (61.5)	
Either parent	259 (59.0)	180 (41.0)	
Others	123 (77.1)	37 (22.9)	
None (living alone)	1 (1.3)	59 (98.7)	
**Currently working**			<0.001^*^
No	669 (41.0)	961 (59.0)	
Yes	274 (56.6)	210 (43.4)	
**School type**			<0.001^*^
Public	382 (45.1)	589 (46.4)	
Private	233 (27.5)	60 (4.8)	
**Receiving nutrition**			<0.001^*^
**education in**			
**schools' curriculum**			
No	453 (52.2)	415 (47.8)	
Yes	211 (53.1)	186 (46.9)	

^*^Significant at *p*-value < 0.001 for χ2 test.

On the other hand, school students had the highest prevalence of low nutrition literacy scores (52.5% of them), exceeding that reported among university students (33.1%), *p* < 0.001. Similarly, more than half of fathers with school (53.9%) or university (52.3%) education levels had adolescent children who were nutritionally literate, with *p*-value < 0.001. However, 52.4% of mothers with a university education level had nutritionally illiterate adolescent children, *p* < 0.001. Additionally, it was observed that adolescents having either parent (mother or father) as a primary caregiver or other caregivers (grandfather, grandmother, other relatives, etc…) had poorer nutrition literacy (59.0 and 77.1% of them, respectively). Likewise, 56.6% of working adolescents were nutritionally illiterate, as opposed to 41.0% of non-workers. Public school students were more nutritionally illiterate than those enrolled in private schools (45.1 vs. 27.5%), *p* < 0.001. Also, 52.2% of adolescents not receiving nutrition education at school had low nutrition literacy scores, compared to 53.1% of adolescents who reported receiving nutrition education in their schools' curriculum, *p* < 0.001 (Table 4).

### 3.5. The determinants of adolescents' nutrition literacy: The binary logistic regression analysis

Based on the bivariate analysis shown above, the author attempted to identify the most significant determinants predicting the adolescents' nutrition literacy status using the backward stepwise method of the binary logistic regression. Table 5 shows that male adolescents, compared to females, were 2 times more likely to have poor nutrition literacy scores (OR = 2.0, CI = 1.5–2.5, *p* < 0.001). Northern border residents were 14 times more probable to be nutritionally illiterate, in contrast to adolescents residing in Riyadh (OR = 14.0, CI = 7.3–28.0, *p* < 0.001). As for weight status, being underweight increased the chance of having poor nutrition scores among adolescents by 3 times (OR = 2.7, CI = 1.6–4.7, *p* < 0.001). As well, overweight adolescents had 2 times more likelihood of being nutritionally illiterate (OR = 2.2, CI = 1.7–2.9, *p* < 0.001).

**Table 5:** The determinants of adolescents' nutrition literacy: The backward stepwise method of the binary logistic regression.

**Binary logistic regression taking the nutrition literacy (TNL) [Poor (reference) vs. Adequate] as the dependent variable**	**AOR (95% CI)**	***p*-valve**
**Gender (Reference: Girls)**	-	-
Boys	2.0 (1.5–2.5)	<0.001^**^
**Residence (Reference: Riyadh)**		
Makkah	0.6 (0.4–0.9)	0.02^*^
Northern borders	14.0 (7.3–28.0)	<0.001^**^
Asir	0.5 (0.1–0.4)	<0.003^*^
Najran	0.2 (0.1–0.4)	<0.001^**^
**Weight status (Reference: Normal weight)**	-	-
Underweight	2.7 (1.6–4.7)	<0.001^**^
Overweight	2.2 (1.7–2.9)	<0.001^**^
**Adolescents' education (Reference: University level)**		
School level	1.8 (1.4–2.4)	<0.001^**^
**Maternal education level (Reference: University level)**		
Illiterate	0.1 (0.03–0.11)	<0.001^**^
School level	0.6 (0.5–0.8)	<0.001^**^
**Paternal education level (Reference: University level)**		
Illiterate	2.4 (1.1–5.0)	0.02^*^
School level	0.8 (0.6–1.1)	0.23
**Primary caregiver (Reference: Both parents)**		
Other caregivers	3.9 (2.2–6.9)	<0.001^**^

Variables entered in step 1: adolescence stage; gender; residency; weight status; adolescents' education level; maternal education level; paternal education level; primary caregiver; working status; school type; receiving nutrition education in schools' curriculum; AOR, adjusted odds ratio; CI, confidence interval; ^*^significant at *p*-value < 0.05; ^**^ significant at *p*-value < 0.001.

School students had double the risk of having poor nutrition literacy, in contrast to those who were enrolled in universities (OR = 1.8, CI = 1.4–2.4, *p* < 0.001). Mothers with lower education level (school level or illiterate) had 60.0 and 10.0% lower probability to have nutrition illiterate adolescents, respectively, *p* < 0.001. respectively. On the other hand, non-educated fathers had 2.4 times more probability of having nutritionally illiterate adolescent children (OR = 2.4, CI = 1.1–5.0, *p* = 0.02). As well, adolescents having caregivers else than their parents were 4 times more susceptible to having poor nutrition literacy scores (OR = 3.9, CI = 2.2–6.9, *p* < 0.001) (Table 5).

## 4. Discussion

This cross-sectional study assessed the adolescents' nutrition literacy and its determinants in a large population-based sample. To the best of author's knowledge, it is the first of its kind at the national level, which is significant given the importance of improving nutrition literacy among adolescents in light of rising obesity levels. This study enrolled a convenience sample of 2,115 adolescents aged 10–19 years old and recruited from almost all Saudi provinces. Around 59.7% of the sampled adolescents were school students, with 76.8% enrolled in public schools. Of them, 68.6% were not receiving nutrition education in their schools' curriculum. Study findings showed that nearly half of the adolescents (44.6%) had poor nutrition literacy. Moreover, their nutrition literacy status was associated with adolescents' gender, residency, education level, weight status, primary caregiver, and maternal and paternal education level as well.

The overall scarcity of studies on nutrition literacy topic makes it a challenge to compare the results of the present study to those of national or regional studies. Recent review research has shown that only three MENA countries—Lebanon, Palestine, and Iran—have discussed nutrition literacy. The review emphasized that people in these nations typically had inadequate nutrition literacy levels. The findings of the current study are comparable to those reported among Lebanese, Palestinian, and Iranian adolescents, in which 44.6% of Saudi Arabian adolescents were found to be nutritionally illiterate (28). Additionally, findings of this study are similar to that reported among Turkish adolescents where a considerable proportion of them had inadequate nutrition literacy levels (32, 33). As well, 51.9% of Chinese adolescents recruited from grades 7,8,10, and 11 at 239 schools in Chongqing were observed to have poor nutrition literacy scores (34). However, a study in North Central Florida, United States showed that adolescents had sufficient levels of nutrition knowledge and literacy (7).

Noteworthy is the fact that research has found that adolescents populations in developed nations tend to have higher levels of nutrition literacy than their counterparts in developing ones. The latter observations were explained by the fact that developed countries had made greater investments in the invention of nutrition education policies for use in and outside the school environment (28). In the same manner, the observed findings could be justified by the fact that 68.6% of Saudi adolescents reported not getting nutrition education in schools. This is supported by findings from recent national research that found no nutrition education programs were offered at any of the 10 elementary schools in Saudi Arabia that participated in the study. More than half the students (55.8%) also stated that they were not taught any classes about nutrition or healthy diets. Also of interest, 64% of teachers in Riyadh, Saudi Arabia lacked access to adequate nutrition curricular materials, and 70% were not trained adequately in nutrition education (35).

Not only do schools fail to educate their students about nutrition and eating healthy, but their canteens also sell unhealthy food. One study found that the diet provided to high-school students in Makkah, Saudi Arabia, was high in sugar and sodium and deficient in calcium, iron, and vitamin D, which is cause for concern (36). Most school food service supervisors in the investigated schools were not trained in nutrition, yet they were responsible for ordering food and creating meal plans (31). Multiple studies (26, 37, 38) confirmed these results by revealing that Saudi adolescents have poor dietary practices and food preferences. Of the 1,133 high school students surveyed, only 27.7% ate breakfast daily on average. Even more concerning, only almost three-fifths of adolescents (35.7%) and slightly more than a quarter (28.6%) said they ate vegetables and fruits every day. Also, more adolescents (37.1%) reported drinking fizzy beverages every day (26).

Lack of awareness of healthy food and unsupportive environment for healthy eating for adolescents may have an impact on the emergence of unhealthy eating habits. As a result, overweight and obesity have often been noted as serious health issues among them, according to plenty of evidence (37–39). The aforementioned assumption may explain the high percentage (38.6%) of adolescents who are currently categorized as overweight or obese, as demonstrated by the current study. Despite the genetic predisposition to obesity, other pliable factors, such as nutrition literacy, may indeed contribute to the prevalence and burden of obesity. Referring to the socio-ecological model (SEM), the cumulative effects of individual, interpersonal, institutional, community, social and political factors affect the adolescent's health and nutrition status. SEM moves beyond solely focusing on individual's (adolescent's) risk factors; however, it represents the multi-level determinants of health (40).

Given the preceding debates, it is now crucial to demonstrate the courage necessary to create supportive school environments in the Kingdom of Saudi Arabia, generating favorable possibilities for nutrition education and enhancing students' nutrition literacy. The Centers for Disease Control and Prevention (CDC) recommends that schools create rules that encourage children to make healthy food choices. For behavior modification, the CDC recommends 40–50 h of nutrition education in this context. To intervene in the nutrition knowledge and skills of kids from diverse socioeconomic backgrounds and with varying dietary demands, schools are the best and most convenient places to do so. Sending out text messages with nutrition-related content or conducting group cooking sessions are two examples of how nutrition education could reach more people outside of the classroom setting (41). Improvements in eating behaviors, label reading, consumption habits, academic achievement, variety in the diet, and nutrient sufficiency have all been linked to higher levels of nutrition literacy in the MENA area (28). So, the school-based nutrition education method will help Saudi Arabia in various ways, such as strengthening the country's food security and addressing the underlying causes of adolescent malnutrition. There has to be strong support from policymakers, nutritionists, health promoters, and other stakeholders for equipping schools with the tools they need to implement effective nutrition education programs.

This study also shows that some factors, such as adolescents' gender, residence, education level, weight status, and the education level of their parents, are important in laying the foundation for their nutrition literacy. This finding is in line with previous research that linked dietary literacy with socioeconomic factors (14, 42, 43). Supporting present study findings, an Iranian study found that being female was a significant predictor of higher levels of nutrition literacy scores. Those females with higher nutrition literacy were more likely to use food labels, buy low-calorie foods, and pay attention to nutrition information. In addition, those who knew more about nutrition also ate less fried chicken and high-fat cheese (42). Furthermore, a similar study found that girls had better levels of functional nutrition literacy than boys (15). The gender of adolescents was also found to significantly correlate with their levels of nutrition literacy in a cross-sectional study of 803 adolescents aged 10–12 from elementary schools in Tehran city, Iran (44). A possible explanation for the disparity in nutrition literacy between male and female adolescents is that girls are more aware of their eating patterns due to concerns about their body image and ideal weight (45). Differential body image perception between the sexes has been documented in research. Girls, even at a young age, are more self-aware about how their weight affects their looks than boys. In addition, being overweight has a greater negative impact on females' self-esteem than on males' (46). This finding suggests that gender-specific approaches to nutrition education are necessary to meet the unique needs of female students without fostering negative body image or an unhealthy preoccupation with food while also aiming to pique boys' interest in nutrition and inspire them to learn more about it.

There was a 3 fold and 2 fold increase in the chance of nutritional illiteracy among those who were underweight and overweight, respectively in the current study. There is a lack of consistency among the studies that have looked at how BMI and nutrition literacy are associated (35, 36), and the results may vary according to age and gender (34, 42, 44, 47–49). Overall, those in their late adolescence stage (17–19 years old) and females were more prevalent in the current study. This result is assumed as adolescents' body weight is determined by a variety of interrelated external factors, such as eating patterns, food preferences, and dietary patterns (50), all of which are founded on the level of nutrition literacy. When it comes to adolescent nutrition in terms of its diversity and sufficiency, NL plays an important role. Adolescents with higher FNL are also less likely to be overweight or obese than those with lower FNL (17). Fried meals, sugary drinks, red meat, and processed foods are more common among individuals with low NL, whereas vegetables, olive oil, and nuts are more common among those with high NL (51). Additionally, a study shows that improved energy balance, lower sugar intake, and higher dairy intake are all connected with higher FNL, which in turn has a beneficial effect on adolescents' weight. Previous studies have linked high INL with higher energy scores, and high CNL with more fruit and vegetable consumption (18). Hence, investments in nutrition literacy, as previously discussed, are essential to break the intergenerational cycle of malnutrition in our nation, as obesity in adolescence is most likely to persist into adulthood. A systematic review and meta-analysis showed that nearly 55% of obese children would become obese in adolescence, 80% of obese adolescents will remain obese in adulthood, and 70% will be obese after the age of 30 (52).

Parental-related factors, including the education level and the primary caregiver, were also shown to contribute to determining adolescents' nutrition literacy level in the present study. In this regard, one study showed that low-educated parents typically have children with unhealthy eating habits (53). The family structure that a child grows up in will actively participate in developing and supporting behaviors that will stick with him or her throughout their life. Parental food habits and feeding strategies are the most dominant determinants of a child's eating behavior and food choices (54). Parents actively choose what to eat for the family, act as role models for dietary choices and patterns, and use feeding practices to bolster the development of eating patterns and behaviors that they find appropriate. According to studies, children's consumption of milk, fruits, and vegetables increased when they witnessed adults eating these meals (55). Parents are undoubtedly the first-ever teachers for their children; therefore, it is fundamental to include parents and adolescents in nutrition education sessions and activities for all planned school-based interventions in Saudi Arabia and other countries.

There were several of policy implications from this study. The economic, social, and health costs associated with obesity are significant. Regarding the prevention and control of NCDs, Saudi Arabia has made some progress in the right direction with regard to the national dietary goals (11). To achieve the 2025 global nutrition goals, Saudi Arabia focused on preventing an increase in childhood obesity and on reducing the prevalence of non-communicable illnesses by reducing the prevalence of their risk factors (56). Children in Saudi Arabia under the age of five are 6.1% overweight, and yet the country has made limited progress in reducing this rate. Few progress has been made toward their aim of preventing obesity, with an estimated 34.3% of adult men and 45.5% of adult women being obese (56). It's predicted that the rate of adolescent obesity in Saudi Arabia would keep climbing in the future years. This assumption is supported by data showing that Saudi adolescents frequently eat at fast food restaurants, rarely eat breakfast, and eat very little fruits and vegetables (25).

Policymakers, researchers, and other stakeholders in society would do well to assess and develop the NL of adolescents because doing so may improve their weight status by improving their ability to make food choices, perceive food labels, implement food safety precautions, apply healthy cooking methods, and adopt appropriate dietary recommendations (57). The environment of the schools might be an excellent starting point for teaching adolescents about their food and the appropriate dietary patterns and eating behaviors.

### 4.1. Study limitations and strengths

The findings of the current study should be interpreted with caution as there are some study limitations. Similar to all cross-sectional design-based studies, it is inappropriate to infer causal relationships between the study variables. In addition, the online self-administered could lead to some bias in the information collected from adolescents as the nutrition literacy topic is still emerging in our region, and it could be more helpful to conduct face-to-face interviews with the adolescents to fill in the data more accurately. Nonetheless, online surveys save time and do not require logistical planning. Moreover, assessments of adolescents' eating habits or diet quality were not performed, although they might associate with the nutrition literacy levels of adolescents. On the other hand, to the best of the author's knowledge, this study is the first of its kind in our nation and could serve as an inspiration for future school-based interventions which should cover adolescents and their parents, particularly in school settings.

## 5. Conclusions

In conclusion, the NL among Saudi Arabian adolescents was investigated here for the first time using a group-specific, validated instrument. The author found that a large percentage of Saudi adolescents demonstrated poor nutrition literacy. Additionally, adolescent's gender, place of residence, weight status, educational level, primary caregiver, or parents' level of education were found to influence NL. These findings suggested that programs targeting NL adolescents could help bring down the risk of obesity. When people make better eating choices and engage in regular physical activity, they reduce their risk of becoming overweight or obese. Adolescence is an important time for forming dietary patterns, which are influenced by a variety of factors including an understanding of nutrition. To equip adolescents and school-age children with the sufficient nutrition knowledge and skills essential to encourage healthy eating decisions, nutrition literacy is one of the most flexible elements that may be perfectly intervened in school settings. Without a doubt, this calls for a holistic strategy on the part of education and health ministries, nutrition professionals, educators, parents, and eager, motivated teenagers. Mechanisms, like the impact of NL on food consumption and exercise levels, should be evaluated in future research.

## Data availability statement

The datasets presented in this study are not readily available due to confidentiality reasons, requests to access the data can be directed to the author KB, kbookari@taibahu.edu.sa.

## Ethics statement

The studies involving human participants were reviewed and approved by the Ethical Committee at Taibah University, Saudi Arabia. Written informed consent to participate in this study was provided by the participants' legal guardian/next of kin.

## Author contributions

KB conceived the study, conducted the analyses, interpreted the results, wrote the paper, critically reviewed the manuscript, and approved the final version submitted for publication.
